# Supplementary material for: Laboratory layered latte
Source: Nat Commun. 2017 Dec 12;8:1960. doi: 10.1038/s41467-017-01852-2 (PMC5727143; doi:10.1038/s41467-017-01852-2)
Supplement: Supplementary file 1 — Supplementary Information [file 41467_2017_1852_MOESM1_ESM.pdf]

SUPPLEMENTARY INFORMATION FOR LABORATORY LAYERED LATTE

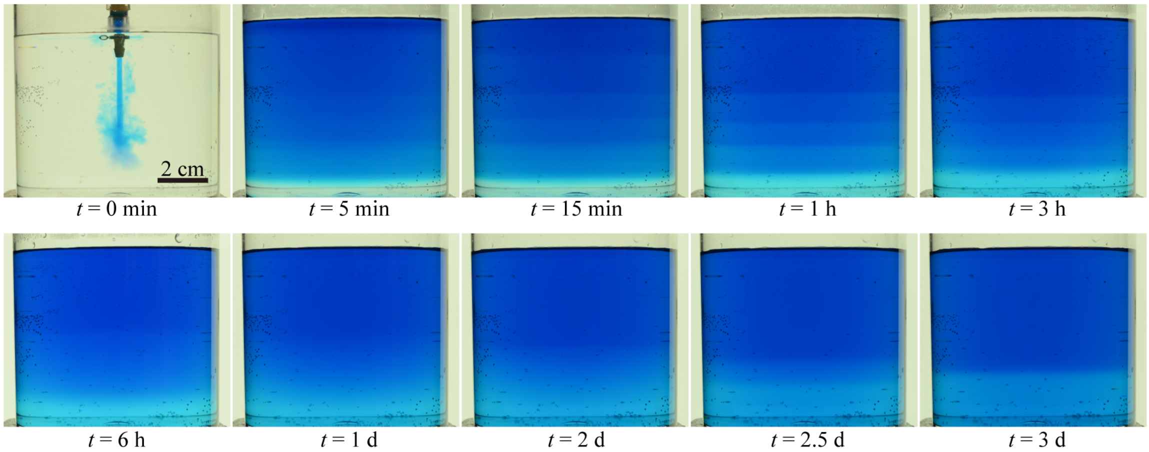

Supplementary Figure 1: Evolution of the layering process after injection of dyed water into brine solution. 30 ml of dyed water is injected into 340 ml of 9.1 wt% sodium chloride solution with injection velocity $U = 0.37$ m s^{-1} at temperature $T_{\mathrm{H}} = 40°$C. The liquid in the tank is cooled at room temperature. Horizontal layers appear within the first five minutes after the injection ($t = 5$ min) and become clearer at $t = 15$ min. As the temperature of the mixture reaches that of the room, the thermal convection decays. The layers eventually merge and form thicker structures, thus the clear boundaries between the initial layers become blurred. These layers last for 2 days ($t = 2.5$ d) before being eliminated by diffusion ($t = 3$ d).

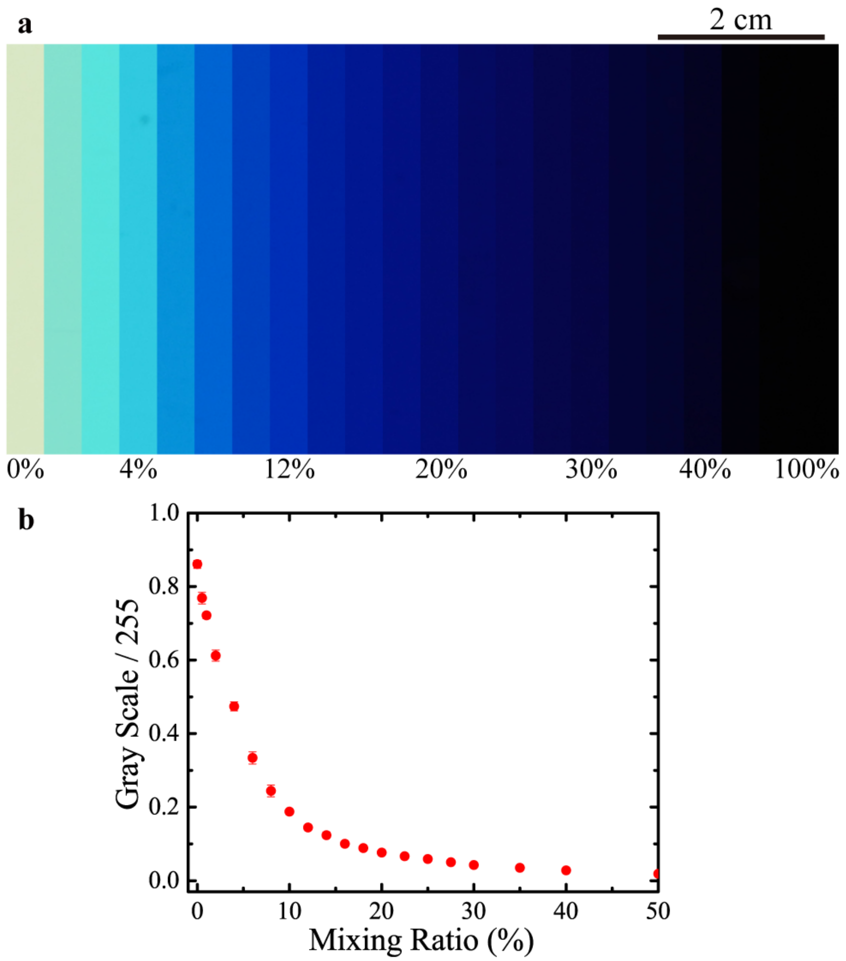

Supplementary Figure 2: Calibrating the fluid density with the light intensity in the digital images. **a** Vertical stripes (50 (width) $\times$ 500 (height) pixels) covering the central region of the tank in the images were considered to determine the average grey-scale intensity in the converted images. Stripes correspond to the images of solutions with different mass concentration ratios c. **b** The average light intensity of the pixels in each stripe is correlated to the density of the mixture according to $\rho = c\rho_{\rm w} + (1-c)\rho_{\rm s}$, where $\rho_{\rm w}$ is the density of water and $\rho_{\rm s}$ is the density of the salt water initially in the tank.

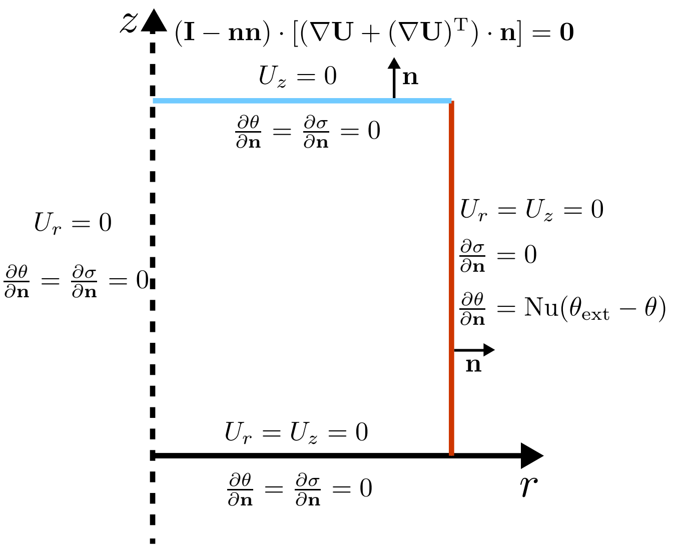

Supplementary Figure 3: Computational domain in the (r, z) cylindrical coordinates and the boundary conditions for the nondimensional velocity $\mathbf{U}$, temperature θ and salinity σ. Here $\mathbf{n}$ denotes the normal vector (of the boundaries) pointing towards the exterior domain.

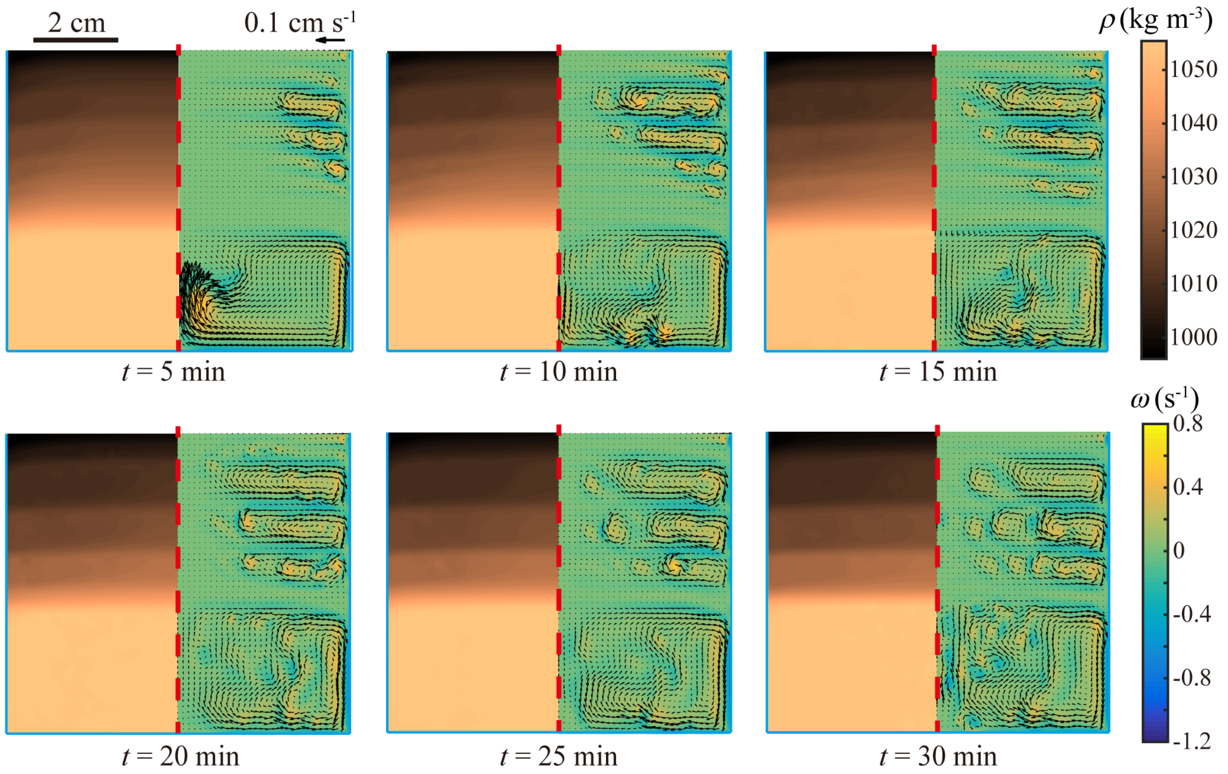

Supplementary Figure 4: Time series of layering in simulations such as those performed for Fig. 2h, i. The vertical dashed line refers to the cylindrical axis. The density distribution is displayed on the left while the velocity vectors and the vorticity magnitude are displayed on the right. Different times $t = 5$, 10, 15, 20, 25 and 30 minutes are chosen to demonstrate the development of layering.

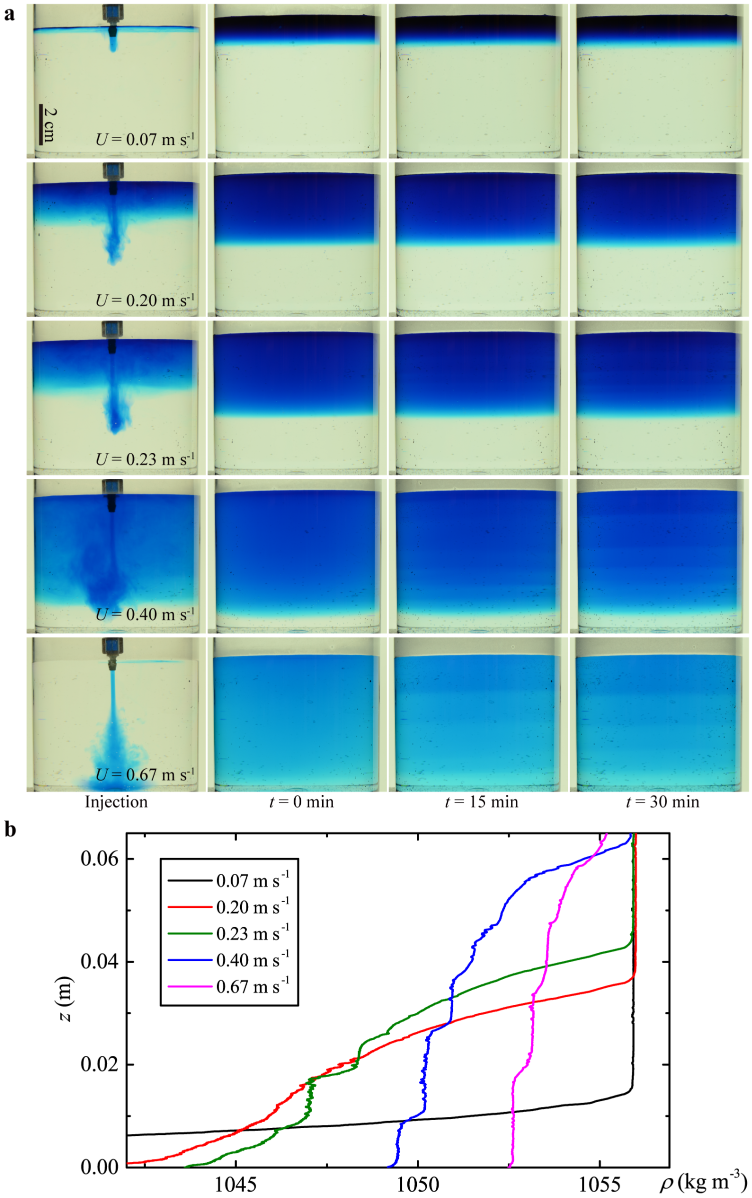

Supplementary Figure 5: Effect of injection velocity on the formation of the horizontal layers. **a** 30 ml dyed water is injected into 340 ml 9.1 wt% sodium chloride solution with different injection velocities $U = 0.07$, 0.20, 0.23, 0.40 and 0.67 m s^{-1}. By increasing the injection velocity, both the injection depth and the thickness H of the mixed volume increase. Layers are observed in the mixture as long as U exceeds the critical injection velocity $U_\mathrm{c} \approx 0.21$ m s^{-1}, while no layers are observed for $U < U_c$. **b** Density profile of at $t = 30$ min. The solid lines from left to right refer to $U = 0.07$, 0.20, 0.23, 0.40 and 0.67 m s^{-1}, respectively. Vertical steps indicate layering occurs.

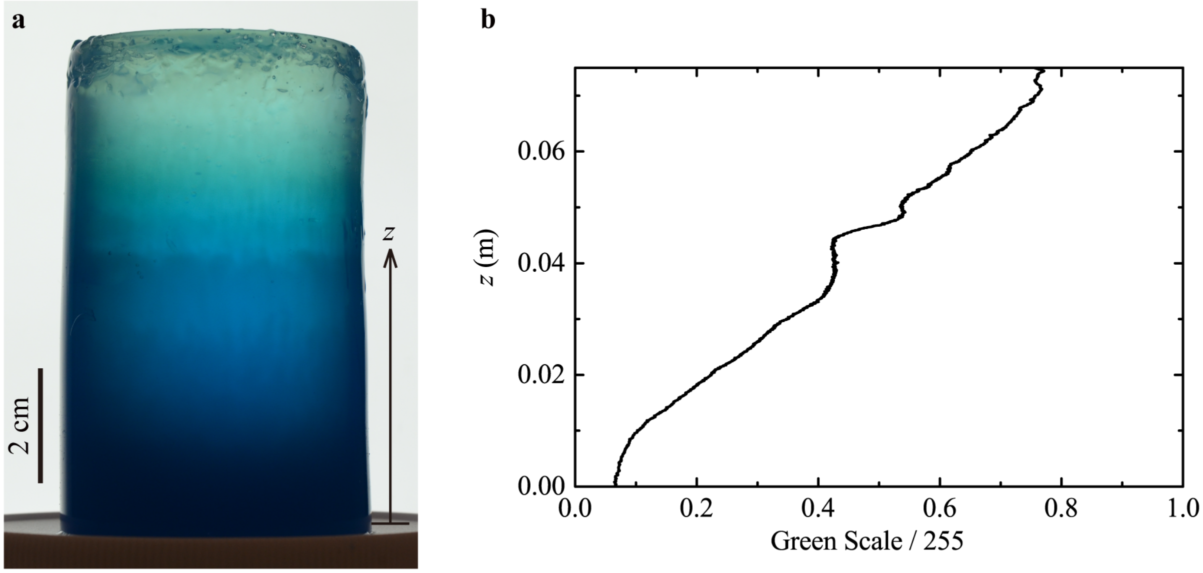

Supplementary Figure 6: **a** Soft layered agarose gel. Horizontal layers are created from an agarose solution in a single-step process by injecting 100 ml 4 wt% agarose solution with 0.0015 wt% methylene blue hydrate into 100 ml 9.1 wt% NaCl solution (without fluorescein sodium salt). **b** Green scale extracted from **a**, plotted versus z. Vertical steps indicate layering in the gel.

SUPPLEMENTARY DISCUSSION

Long-lasting layered patterns

The temperature of the heat source (the liquid in the tank) in our experiments decreases gradually until reaching room temperature. At this point no significant thermal gradient is present in the mixture to support the layered patterns and distinct patterns in the mixture eventually disappear due to diffusion. In our experiments with dyed salt solutions, the layered structures vanished after 3 days (Supplementary Fig. 1).

Effect of injection velocity

The injection velocity U affects the density profile and so the density gradient in the mixture and consequently the formation of the layered patterns. At low injection velocities $U < U_c$, no layers are formed and no steps are obtained in density profile, while increasing U results in the appearance of distinct layers and vertical steps are obtained in the density profile (Supplementary Fig. 5).

Quantification of the density gradient

A calibration procedure is performed to correlate the local intensity of the blue dye in the digital images to the local concentration c (mass ratio) of injected dyed water containing 0.01 wt% methylene blue hydrate. For the calibration, we prepare solutions with different mass concentration ratios $0 \leq c \leq 1$. The mass ratio of the solution is controlled by mixing $c \times 100$ wt% dyed water (water with 0.01 wt% methylene blue hydrate, as used in model experiments) with $(1 - c) \times 100$ wt% brine (9.1 wt% sodium chloride solution, as used in experiments). The final solution is placed in a tank similar to that used in the model experiments and color images of the solution are captured with back-lit illumination intensity identical to that employed in the model experiments. A vertical stripe (50 (width) $\times$ 500 (height) pixels) covering the central region of the tank in the image is used to determine the average grey-scale intensity in the converted images. Supplementary Fig. 2a shows such stripes obtained from images of solutions with different mass concentration ratios c. Based on this information, we calibrate the local intensity of the blue dye to obtain the local mass ratio of injected dyed water in the mixture, and then calculate the local density in the mixture considering $\rho = c\rho_{\mathrm{w}} + (1-c)\rho_{\mathrm{s}}$, where ρ_{w} is the density of water and ρ_{s} is the density of the salt water initially in the tank. Supplementary Fig. 2b demonstrates the correlation between the average gray scale intensity obtained from the images as illustrated in (a) and

the mass concentration ratio c.

Transient numerical simulations

A schematic of the domain used in the numerical simulations is presented in Supplementary Fig. 3. Results of our transient numerical simulations demonstrate the development of layering in time (Supplementary Fig. 6). The axisymmetric flow circulations in the convection cells are clockwise in the right side of the tank. Smaller secondary circulating flows in a given layer merge in time to form a more elongated cell in the horizontal direction. The magnitude of velocities in the circulation zones is higher in the middle-top region, where the density gradient is smaller according to the density profile (Fig. 2e), resulting in stronger mixing and more distinct layers. The density gradient is larger near the bottom of the mixture, which leads to a nearly stationary flow in this region. The original brine (with no salinity gradient) occupies the bottom of the tank, where normal thermal convection occurs as a result of cooling from the side wall of the tank.

Layered soft agarose gel

We make multiple layers in an agarose gel simply by a single-step of injecting ($U \approx 1$ m s$^{-1} > U_{\mathrm{c}}$) a hot gel solution into a denser solvent and cooling the mixture at room temperature. When cooling, the layers form in the agarose solution and then the solution solidifies to a layered gel when below the gelation temperature. To prove the existence of layering in gel, we use only methylene blue hydrate for visualization. We extract the green scale in the gel image and plot the green scale versus the position z. The green scale varies from the bottom (darker, harder) to the top (lighter, softer) and at least two vertical steps in the plot indicate layering occurs in the gel.

SUPPLEMENTARY METHODS

The layered color gel is fabricated with the following recipe: 1. Prepare 100 ml 4 wt% agarose-water mixture with 0.0015 wt% methylene blue hydrate and 100 ml 9.1 wt% NaCl solution containing 0.002 wt% fluorescein sodium salt (fluorescein sodium salt is a yellow dye and is chosen to be distinguished from the blue dye). 2. Heat the agarose-water mixture and the dyed NaCl solution to reach $T = 95$ °C. 3. Then cool the mixture down to 80°C and pour the agarose solution into the NaCl solution. 4. Finally, the whole mixture solidifies in a fridge where $T = 0$ °C for one hour to form the layered colorful gel.
