# Supplementary material for: Laboratory layered latte
Source: Nat Commun. 2017 Dec 12;8:1960. doi: 10.1038/s41467-017-01852-2 (PMC5727143; doi:10.1038/s41467-017-01852-2)
Supplement: Supplementary file 3 — Description of Additional Supplementary Files [file 41467_2017_1852_MOESM3_ESM.pdf]

Description of Additional Supplementary Files

File Name: Supplementary Movie 1
Description: The Supplementary Movie for layering by pouring warm espresso into warm milk.
